# Data-driven multimodal fusion: approaches and applications in psychiatric research

**DOI:** 10.1093/psyrad/kkad026

**Published:** 2023-11-22

**Authors:** Jing Sui, Dongmei Zhi, Vince D Calhoun

**Affiliations:** State Key Laboratory of Cognitive Neuroscience and Learning, Beijing Normal University, Beijing 100875, China; State Key Laboratory of Cognitive Neuroscience and Learning, Beijing Normal University, Beijing 100875, China; Tri-institutional Center for Translational Research in Neuroimaging and Data Science (TReNDS), Georgia Institute of Technology, Emory University and Georgia State University, Atlanta, GA 30303, United States

**Keywords:** multimodal fusion approach, data driven, functional magnetic resonance imaging (fMRI), structural MRI, diffusion magnetic resonance imaging, independent component analysis, canonical correlation analysis, psychiatric disorder

## Abstract

In the era of big data, where vast amounts of information are being generated and collected at an unprecedented rate, there is a pressing demand for innovative data-driven multi-modal fusion methods. These methods aim to integrate diverse neuroimaging perspectives to extract meaningful insights and attain a more comprehensive understanding of complex psychiatric disorders. However, analyzing each modality separately may only reveal partial insights or miss out on important correlations between different types of data. This is where data-driven multi-modal fusion techniques come into play. By combining information from multiple modalities in a synergistic manner, these methods enable us to uncover hidden patterns and relationships that would otherwise remain unnoticed. In this paper, we present an extensive overview of data-driven multimodal fusion approaches with or without prior information, with specific emphasis on canonical correlation analysis and independent component analysis. The applications of such fusion methods are wide-ranging and allow us to incorporate multiple factors such as genetics, environment, cognition, and treatment outcomes across various brain disorders. After summarizing the diverse neuropsychiatric magnetic resonance imaging fusion applications, we further discuss the emerging neuroimaging analyzing trends in big data, such as N-way multimodal fusion, deep learning approaches, and clinical translation. Overall, multimodal fusion emerges as an imperative approach providing valuable insights into the underlying neural basis of mental disorders, which can uncover subtle abnormalities or potential biomarkers that may benefit targeted treatments and personalized medical interventions.

## Introduction

The escalating prevalence of psychiatric disorders has imposed a substantial economic burden on society (Ferrari *et al*. 2022), particularly exacerbated by the impact of the COVID-19 pandemic. Compelling evidence has suggested that the presence of psychiatric disorders is associated with altered brain function and structure, and magnetic resonance imaging (MRI) has emerged as a non-invasive technique with significant promise for investigating brain changes. Currently, collecting multiple types of non-invasive brain imaging data from the same individual has become a common practice, aiming to identify potentially stable task- or disease-related changes and thus, to improve the translation of research findings into clinical practice. Each imaging technique provides a unique perspective of brain function or structure, such as functional MRI (fMRI) for hemodynamic response related to neural activity in the brain, electro-encephalography (EEG) for electrical activity with higher temporal but lower spatial resolution than fMRI, structural MRI (sMRI) for brain tissue type, as well as diffusion MRI (dMRI) for tissue microstructure and brain connectivity. Despite separate analysis of each data modality can provide important insights into the brain structural or functional integrity associated with physiological or behavioral features, there is increasing evidence that multimodal brain imaging can offer a better understanding of inter-subject variability from how brain structure shapes brain function, to what degree brain function feeds back to change its structure, and what functional or structural aspects of physiology ultimately drive cognition and behavior (Sui, Adali, *et al*., 2012; Sui, Huster, *et al*., 2014). Consequently, a key motivation for jointly analyzing multimodal data is to leverage the cross-information in the existing data, thereby potentially revealing important variations that may only be partially detected by a single modality.

The availability of multimodal brain imaging allows for joint analysis via the application of various data fusion approaches (Calhoun *et al*., 2016), including (i) visual inspection, which is basically inferring the multimodal information by separately visualizing results from essentially unimodal analyses; (ii) data integration, which is analyzing each data type separately and overlay them, thereby not allowing for an examination of interaction among data types (Ardnt, 1996; Savopol *et al*., 2002); (iii) asymmetric fusion, using one dataset to constrain another, such as dMRI being constrained by sMRI or fMRI data, which may impose potentially unrealistic assumptions on the constrained data (Goldberg-Zimring *et al*., 2005; Abramian *et al*., 2021; Behjat *et al*., 2021); and (iv) symmetric fusion, which treats multiple image types equally, taking full advantage of the joint information in multiple datasets and providing more views for individual subjects and co-variation between modalities (Sui, Adali, *et al*., 2012; Sui, Huster, *et al*., 2014). Symmetric fusion approaches can be broadly classified as being either model-based or data-driven. Model-based approaches, such as multiple linear regression, dynamic causal modeling, and structural equation modeling, examine the goodness-of-fit of the data to the prior knowledge about the experimental paradigm and the properties of the data. Despite being widely used in biomedical data analysis, model-based approaches are limited when the dynamics of the experiment become hard to model. However, data-driven methods are suitable for the analysis of such complex paradigms as they minimize the assumptions on the underlying properties of the data by decomposing the observed data based on a generative model. Data-driven approaches include, but are not limited to, principal component analysis (PCA), independent component analysis (ICA), and canonical correlation analysis (CCA). These methods belong to blind source separation approaches, as they do not require prior hypotheses about the connection of interest; hence, they are attractive for the exploration of the full body of data. We have developed several data fusion approaches based on ICA and CCA (Sui, Adali, *et al*., 2012; Qi, Calhoun, *et al*., 2018), and applied them to unravel intricate relationships among genetic, brain imaging, and behavior, aiming to elucidate the complex neural mechanisms underpinning various psychiatric disorders and to facilitate personalized clinical interventions.

In this paper, we first provide some basic motivation regarding the benefits of data-driven multimodal fusion and introduce some basic terminology for characterizing multimodal data analysis. Next, we provide a summary of multivariate approaches for multimodal data fusion, with an emphasis on ICA- or CCA-based methods. Following this, we review existing studies that have used multimodal fusion approaches to investigate psychiatric disorders, and whenever possible, the behavioral relevance of the assessed physiological features will be mentioned. Finally, we discuss some emerging trends and approaches.

## Data-driven fusion approaches using multimodal MRI

Different brain imaging data types are intrinsically dissimilar, making it difficult to analyze them together without making several assumptions. Instead of directly analyzing the entire datasets together, an alternate approach is to reduce each modality to a feature, a distilled dataset representing the interesting part of each modality (Calhoun *et al*., 2009), such as the fractional amplitude of low-frequency fluctuations (fALFF) from fMRI, fractional anisotropy (FA) from dMRI, or segmented gray matter (GM) from sMRI, providing a natural way to discover multimodality associations and also alleviating the difficulty of fusing data types of different dimensionality and nature, as well as those that have not been recorded simultaneously. The trade-off is that some information may be lost, e.g. GM does not directly measure volume or cortical thickness and FA does not provide directional information. Nevertheless, there is considerable evidence supporting the usefulness and validity of feature-level analysis (Smith *et al*., 2009). By contrast, emerging fusion approaches have been developed to directly handle the first-level 4D fMRI data to extract individual variations from high-dimensional raw images by integrating multi-level PCA and subject-level back-reconstruction techniques (Du *et al*., 2013; Qi *et al*., 2019).

**Figure 1:**
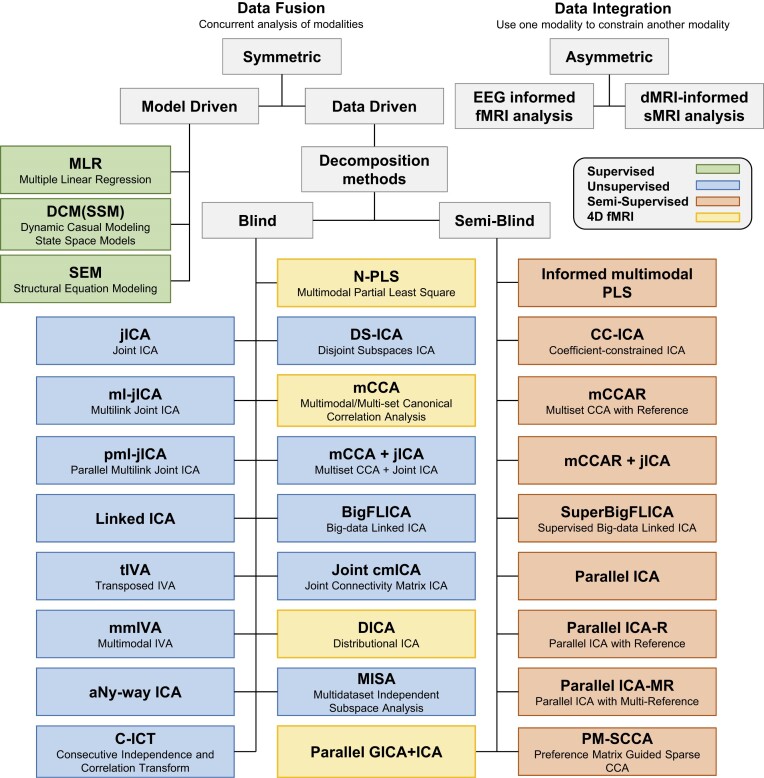
Overview of the current popular multimodal fusion approaches. The fusion models with model-driven (green), unsupervised (blue), and semi-supervised (orange) data-driven learning are listed in different categories, in which the models that can deal with 4D fMRI data are highlighted in yellow.

Here we review the multivariate fusion methods due to their flexibilities and advantages, with an emphasis on ICA- and CCA-based approaches. Based on the requirement of *a priori* and the dimension of the fMRI data used, the data-driven multivariate approaches adopted in multimodal MRI fusion can be further divided into three classes (Fig. 1):

- Blind methods that typically use second-level fMRI data (3D contrast image), including joint ICA (jICA), disjoint subspace ICA (DS-ICA) (Adali *et al*., 2018), multilink jICA (ml-jICA) (Khalilullah *et al*., 2023), and parallel ml-jICA (pml-jICA) (Khalilullah *et al*., 2023), multimodal CCA (mCCA) (Correa *et al*., 2007; Sui *et al*., 2015), mCCA + jICA (Sui *et al*., 2011; Sui, He, *et al*., 2012), linked ICA (Groves *et al*., 2011), big-data linked ICA (BigFLICA) (Gong *et al*., 2021), transposed independent vector analysis (tIVA) (Adali *et al*., 2015), multimodal IVA (Damaraju *et al*., 2021), aNy-Way ICA (Duan *et al*., 2020), and consecutive independence and correlation transform (C-ICT) (Jia *et al*., 2021), and multidataset independent subspace analysis (MISA) (Silva *et al*., 2020).
- Blind methods that have been developed for raw fMRI data (4D data), including partial least squares (Chen *et al*., 2009), multiset CCA (Correa *et al*., 2010), distributional ICA (Wu *et al*., 2021), and joint connectivity matrix ICA (joint cmICA) (Wu *et al*., 2023).
- Semi-blind methods that use second-level fMRI data (3D contrast), e.g. parallel ICA (pICA) (Liu *et al*., 2009), pICA with reference (pICA-R) (Liu *et al*., 2012), pICA with multiple references (pICA-MR) (Chen *et al*., 2014), coefficient-constrained ICA (Sui, Adali, Pearlson and Calhoun, 2009; Sui, Adali, Pearlson, Clark, *et al*., 2009), PCA with reference (Caprihan *et al*., 2008; Liu *et al*., 2008), informed multimodal partial least squares (Chen *et al*., 2009), multiset CCA with reference (mCCAR), mCCAR + jICA (Qi, Calhoun, *et al*., 2018), and preference matrix guided sparse CCA (PM-SCCA) (Sha *et al*., 2023). Moreover, the most recent parallel group ICA + ICA is able to deal with 4D fMRI and 3D sMRI features simultaneously (Qi *et al*., 2019).

## Review of multivariate, multimodal fusion models

In the following sections, we will introduce several blind fusion models and semi-blind models, as well as discuss their characteristics in combining multimodal neuroimaging.

### Joint ICA

jICA is one of the ICA-based data fusion methods that jointly analyze multiple datasets by concatenating them along a certain dimension (Calhoun, Adali, Kiehl, *et al*., 2006). jICA is based on the assumption that two or more features share the same mixing matrix and order as well as equal contributions, and maximizes the independence among joint components. jICA is feasible for many paired combinations of features, such as fALFF, GM, FA, or N-way data fusion (Calhoun, Adali and Liu, 2006; Franco *et al*., 2008; Xu *et al*., 2009).

### Multilink jICA

Instead of reducing fMRI into a single map or a single intrinsic connectivity network like default mode network, Khalilullah *et al*. proposed ml-jICA to fuse GM and multiple rest fMRI networks, such as intrinsic connectivity networks, using the same core algorithm as jICA (Khalilullah *et al*., 2023). However, jICA assumes similar distributions among different modalities, and GM maps have a very different distribution from intrinsic connectivity networks derived from ICA, which are already maximally independent (Du *et al*., 2020). Therefore, they further proposed pml-jICA (Khalilullah *et al*., 2023), which allows for a shared mixing matrix for both the sMRI and fMRI modalities, while allowing for different mixing matrices linking the sMRI data to the different intrinsic connectivity networks.

### Disjoint subspace analysis using ICA

The assumption of the same mixing matrix across all modalities in the jICA can be a very constraint, especially with more than two modalities. DS-ICA is introduced to identify and split all modalities into common and their distinct subspaces, and perform separate analyses in subspaces. The order of common and distinct subspaces is determined by the consecutive steps of PCA and CCA. Given the order, the common subspace across all modalities is decomposed with jICA whereas separate ICAs are used in the distinct subspaces (Adali *et al*., 2018; Akhonda *et al*., 2019).

### Joint connectivity matrix ICA

Instead of being based on the selected regions of interest, cmICA decomposes voxel-wise brain connectivity matrix using ICA, generating maximally independent spatial sources and their corresponding connectivity maps to the whole brain (Wu *et al*., 2015). Incorporating the principles of jICA, Wu *et al*. proposed joint cmICA (Wu *et al*., 2023), a data-driven parcellation and automated linking of voxel-wise structural connectivity and functional connectivity information from whole-brain fMRI and dMRI without the need for a prior atlas. The joint cmICA can automatically extract connectivity-based cortical sources that are shared between functional connectivity and structural connectivity, providing more flexibility in estimating sources and connectivity maps.

### Multimodal CCA

mCCA allows a different mixing matrix for each modality and is used to find the linear combinations of variables in each dataset that maximize the inter-subject covariations across datasets, generating a set of components and their corresponding mixing profile, called canonical variants (CVs) (Correa *et al*., 2008). After decomposition, the CVs correlate each other only on the same indices and their corresponding correlation values are called canonical correlation coefficients. Compared to jICA, which constrains features to have the same mixing matrix, mCCA is flexible in that it allows common as well as distinct levels of connection between two features, but the associated source maps may not be spatially sparse, especially when the canonical correlation coefficients are not sufficiently distinct (Sui *et al*., 2010). The mCCA is invariant to differences in the range of the data types and can be used to jointly analyze very diverse data types. It can also be extended to multi-set CCA to incorporate more than two modalities (Li *et al*., 2009). Note that mCCA works on second-level fMRI features, whereas multiset-CCA can also work with 4D raw fMRI data (Correa *et al*., 2010).

### mCCA + jICA

Considering previous findings on multiple modalities (Rykhlevskaia *et al*., 2008; Camara *et al*., 2010), it is plausible to assume that the components decomposed from each modality have some degree of correlation between their mixing profiles among participants. The mCCA + jICA is a blind data-driven model that is optimized for this situation (Sui *et al*., 2011; Sui *et al*., 2013) and also has excellent performance for achieving both flexible modal association and source separation. It takes advantage of two complementary approaches: mCCA and jICA, allowing for both strong and weak connections as well as joint independent components. The mCCA enhances the reliability of jICA by providing a closer initial match via correlation; while the jICA further decomposes the remaining mixtures in the associated maps and relaxes the requirement of sufficient distinction imposed on the canonical correlation. Note that the mCCA + jICA approach does not increase the computational load appreciably and is not limited to two-way fusion, but can potentially be extended to three-way or *N*-way fusion of multiple data types by replacing mCCA with multi-set CCA (Li *et al*., 2009). It enables robust identification of correspondence among N-diverse data types and facilitates investigations into whether certain disease risk factors are shared or distinct across multiple modalities.

### mCCAR + jICA

While maintaining the performance of mCCA + jICA, we hope to optimize specific subject-level correlations with a measure of interest, e.g. cognitive/behavioral score, disease symptom, or genetic variant. Therefore, a supervised, goal-directed model that uses previous information as a reference to guide multimodal data fusion becomes a natural option. To address this, a fusion with reference model called “multi-site CCA with reference + joint independent component analysis” (mCCAR + jICA) was proposed (Qi, Calhoun, *et al*., 2018), which can identify co-varying multimodal imaging patterns associated with the reference with higher estimation precision. The mCCAR + jICA consists of two steps, where the mCCAR was first implemented by imposing an additional constraint to maximize not only the covariations among mixing matrices of each modality, but also the column-wise correlations between mixing matrices of each modality and the reference signals, resulting in the potential target components that are correlated with reference signals in each modality, as well as being most correlated across participants between modalities. jICA is further applied on the concatenated components to keep the modality linkage of the potential target components and maximize the spatial independence, generating the final independent components as well as their corresponding mixing matrices. By incorporating prior information, the mCCAR + jICA enables the identification of joint multimodal components that have robust correlations within referred measures and among themselves (inter-modality correlations) (Qi, Yang, *et al*., 2018; Zhi *et al*., 2020; Qi *et al*., 2021; Xu *et al*., 2022), which may not be detected by a blind *N*-way multimodal fusion approach.

### PM-SCCA

The PM-SCCA is another CCA-based feature-level fusion approach for fusing a vast number of genetic markers, such as single-nucleotide polymorphisms (SNPs), and multimodal quantitative traits, including imaging and cognitive measures. By incorporating prior knowledge, the method takes priors encoded as a preference matrix into a simplified version of sparse CCA to regularize the magnitude of the elements of canonical weight vectors (Sha *et al*., 2023). Therefore, the proposed PM-SCCA model can not only capture multi-SNP-multi-quantitative traits associations, but can also relevant genetic and phenotypic features effectively.

### Linked ICA

Linked ICA is a probabilistic approach based on a modular Bayesian framework, which is designed for simultaneously modeling and discovering common characteristics across multiple modalities (Groves *et al*., 2011). The combined modalities can potentially have completely different units, noise levels, spatial smoothness, and intensity distributions. In linked ICA, each modality is modeled using Bayesian tensor ICA (Beckmann *et al*., 2005) that differs from traditional ICA methods such as fast ICA (Hyvärinen *et al*., 2000) and Infomax (Bell *et al*., 1997) in that it incorporates dimensionality reduction into the ICA itself through automatic relevance determination. Linked ICA can automatically determine the optimal weighting for each modality, and can also detect single-modality structured components when present.

### Big-data linked ICA

Nevertheless, the linked ICA approach encountered computational challenges when dealing with multimodal high dimensional and big sample size datasets, especially the release of international large datasets, such as the UK Biobank (Sudlow *et al*., 2015), ABCD study (Casey *et al*., 2018), and HCP (Van Essen *et al*., 2013) datasets. Consequently, BigFLICA was proposed by integrating MELODIC's incremental group PCA to capture modes with even small variations within each modality (Smith *et al*., 2014) and online dictionary learning (Mairal *et al*., 2010) to reduce the dimension of feature (e.g. voxel) space, which can capture both local and distant spatial correlation structures (Gong *et al*., 2021). BigFLICA can both preserve key information in original data and reduce the effects of stochastic domain-specific noise, as well as increase the computational efficiency of the linked ICA algorithm for extremely large population datasets.

### Semi-supervised BigFLICA

It is evident that both linked ICA and BigFLICA are purely unsupervised learning methods that do not use prior information, such as non-imaging derived phenotypes. Hence, Gong *et al*. introduced a semi-supervised, multimodal, and multi-task fusion approach for IDP discovery, termed semi-supervised BigFLICA (SuperBigFLICA) (Gong *et al*., 2022), which used external phenotype information to guide the identification of relevant multimodal brain networks associated with the phenotype of interest.

### Parallel ICA

The pICA is another ICA-based feature-level fusion approach that can process multiple modalities simultaneously (Liu *et al*., 2009), and uncover the independent components of each modality and the relations among them. The pICA algorithm maximizes the cost function based on both entropy and the correlation term, implemented by identifying the maximally independent components within each dataset individually. Compared to jICA with strong constraints with common mixing matrix and order across all modalities, pICA provides a flexible framework to combine multiple data types with different ranges and properties, such as different neuroimaging, genetic, and phenotypic data. The two- and three-way pICA have been implemented to identify links among genetic, brain structure, and brain function (Vergara *et al*., 2014; Pearlson *et al*., 2015). The pICA has demonstrated superior efficacy in investigating the imaging-genetic associations, and the findings provide proof of concept that genomic SNP factors can be investigated by using phenotypic imaging findings in a multivariate format (Pearlson *et al*., 2015).

### Parallel ICA with reference

No prior gene information is taken into account in the pICA. Nevertheless, incorporating known genes involved in critical biological pathways in disease may help identify a set of genes contributing in a coordinated way to a larger network. Therefore, pICA-R was proposed by imposing an additional constraint on the infomax framework to minimize the distance between a certain component and the reference (Liu *et al*., 2012).

### Parallel ICA with multiple references

A key factor that affects the performance of pICA-R is the reference accuracy. Degradation is expected in component, loading, and linkage accuracies when the reference accuracy is below 0.2. Especially in SNP analysis, a referential SNP set associated with the same trait of interest is desired to obtain a more reliable reference. Therefore, pICA-MR is designed to directly combine multiple referential SNP sets to constrain the component of interest (Chen *et al*., 2014). Compared to pICA-R, this extended approach is more flexible in dynamically constraining components for multiple references and allows for some extent of heterogeneity in references.

### Parallel group ICA + ICA

Many existing multimodal fusion approaches in fMRI focus on 3D feature summaries, neglecting the rich temporal information. Thus, the parallel group ICA + ICA fusion method was proposed to directly deal with the first-level 4D fMRI data (Qi *et al*., 2019; Qi, Silva, *et al*., 2022). This method integrates group ICA into pICA in a unified optimization framework, in which a new variability matrix is defined to capture subject-wise functional variability and used to link the mixing matrices of another modality. Such a method allows two-way fusion of 4D fMRI data with structural MRI features, facilitating the identification of multimodal spatiotemporal links and providing alternative views to investigate brain disorders in a unifying multimodal framework.

### Independent vector analysis

IVA extends ICA to multiple datasets (Lee *et al*., 2008), providing a natural and extendable way to directly link multivariate brain imaging data together. Based on the assumption of independence among sources within each dataset but dependence across datasets, IVA allows for a more flexible way of detecting the dependence across datasets by defining a source component vector that collects the independent components for each dataset, and has shown power in preserving the dataset variabilities when analyzing multiple datasets (Laney *et al*., 2015; Luo, 2023). IVA can be regarded as not only an extension of ICA, but also a generalization of CCA. By integrating higher-order statistics into the mCCA-based model, tIVA was proposed. This approach constrains the statistical independence for independent components within each dataset but statistical dependence across the datasets to fuse different modalities (Adali *et al*., 2015). Moreover, multimodal IVA was implemented by using MISA in the IVA model, thereby identifying common independent sources among multiple modalities (Damaraju *et al*., 2021).

### aNy-way ICA

Whereas most fusion approaches require the same number of sources and/or components for all modalities (jICA, mCCA, mCCA(R) + jICA), Duan *et al*. proposed aNy-way ICA by combining infomax ICA and Gaussian IVA (IVA-G) via a shared weight matrix model without orthogonality constraints, which can simultaneously maximize the independence of sources and correlations across different modalities with the same or different numbers of sources per modality (Duan *et al*., 2020). When applied to the fusion of sMRI, fMRI, and EEG with different numbers of sources, this approach is able to recover sources and loadings, as well as the true covariance patterns with improved recovery accuracies compared to mCCA and mCCA + jICA, especially under noisy conditions.

### Consecutive independence and correlation transform

Existing fusion methods often require the signal subspace order to be identical for all modalities, and cannot discover one-to-many associations, in which one component from one modality is linked with more than one component from another modality. To address this, C-ICT was developed by combining ICA and IVA-G for the joint analysis of multimodal data, including four steps: (i) performing ICA on individual datasets separately; (ii) selecting meaningful ICs and the corresponding subject covariations; (iii) performing IVA-G on the selected subject covariations of different datasets; and (iv) identifying significantly pair-wise associated source component vectors, and tracing back to the ICs in the ICA stage based on subject covariations with the highest contribution to the correlated source component vectors and identify them as associated components across different modalities (Jia *et al*., 2021). C-ICT is flexible in terms of the number of datasets combined and the number of orders of the signal subspace for each dataset, and can discover one-to-many associations.

### Multidataset independent subspace analysis

MISA is a unified multidataset multidiversity multidimensional framework for subspace modeling (Silva *et al*., 2020). In this framework, multiple datasets are jointly decomposed, in which sources are combined into dimensional subspaces that can accommodate arbitrary links among groups of sources across different datasets and modalities, and all-order statistics are used to gauge their associations and pursue subspace independence. Compared with independent subspace analysis that is limited to subspaces within the same dataset, or IVA approaches that have a rigid subspace structure in which a single component (no more, no less) from each dataset must go together to form a subspace, MISA allows for datasets to be not only heterogeneous but also of different dimensionality, combining modalities of different intrinsic dimensionality in a single unified model and providing a robust generalization of many multivariate approaches including ICA, IVA, and independent subspace analysis (Silva *et al*., 2016; Silva *et al*., 2020).

Collectively, each method provides a unique perspective for interpreting the multiple datasets based on their various hypotheses. We summarized and compared the methods in Table 1 on their various optimization assumptions, purpose of the analysis, requirement of priors, number of the modalities, input data types required, and data dimensionality reduction methods, as well as their advantages and disadvantages, aiding in selecting the appropriate fusion method based on the available datasets.

**Table 1:** Summary of assumptions, aims, and suggestions of uses for multimodal fusion methods.

Methods	Optimization assumptions	Goals and purpose	Need of priors	Number of modality	Input data	Dimension reduction	Advantages and disadvantages
jICA	Assume two or more features share the same mixing matrix and order as well as equal contributions. Maximize the independence among joint components.	To examine a commonmixing modulation across subjects among modalities and to find the linked source maps.	No	2 is preferred, 3 + is possible.	Features	PCA	The method is feasible for many paired combinations of features. In contrast, a strong assumption across all modalities may be unrealistic, especially more than two modalities.
pml-jICA	Assume a shared mixing matrix for the fMRI and other modalities, while allowing for different mixing matrices linking the other modality data to the different independent component networks (ICNs). Maximize the independence among multiple ICNs and other modalities.	To detect multiple linked sources for the fMRI and other modalities.	No	2	Features	PCA	The method enables associations between brain function and brain structure from multiple rest fMRI networks, allowing multiple loading sets for each participant.
DS-ICA	Identify and split all modalities into common and distinct subspaces. Maximize the independence among common subspaces using jICA and their distinct subspaces via ICA separately.	To extract linked sources as well as their respective unique independent sources for different modalities.	No	2 + is preferred	Features	PCA	The method is suitable to mine the covarying and unique components simultaneously for different modalities.
Joint cmICA	Assume structural connectivity (SC) and functional connectivity (FC) share the same mixing matrix. Maximize the independence among joint components.	To find the linked connectivity-based cortical sources between SC and FC matrix.	No	2	Features	PCA	The method provides a data-driven parcellation and automated linking of SC and FC information simultaneously.
mCCA	Maximize covariation of the mixing profiles across the two modalities.	To detect common as well as distinct levels of connection between subject modulation.	No	2 is classical 3 + is possible	Features or raw data	PCA	The method is feasible to jointly analyze very diverse data types. However, the associated source maps may not be spatially sparse.
mCCA + jICA	Assume the decomposed components from each modality have some degree of correlation between subject-mixing profiles. mCCA is first used to make the jICA job more reliable by providing a close initial match via correlation; jICA further separates the remaining mixtures in the joint maps.	To achieve both flexible modal association (high or low correlation) and accurate source separation.	No	2 + is preferred	Features	PCA	The method takes advantage of mCCA and jICA, and enables robust identification of correspondence among multiple modalities.
mCCAR + jICA	Maximize covariation of the mixing profiles across different datasets and their correlations with the reference. The jICA further separates the remaining mixtures in the joint maps.	To identify co-varying multimodal imaging patterns associated with the reference.	Yes	2 + is preferred	Features	PCA	The method enables the identification of joint multimodal components that have robust correlations within referred measures and among themselves.
PM-SCCA	Maximize covariation of the mixing profiles across different datasets and their correlations with prior information.	To detect associations between multiple SNP and multiple quantitative traits.	Yes	2	Features	No	The methods can not only take prior information but also maintain computational simplicity.
Linked ICA	All modalities share the same subject-mixing matrix, while each modality (group) is modeled as a sum of components using a tensor decomposition (Bayesian tensor ICA). Modalities with similar spatial properties can be grouped into one modality group, sharing the same source maps as well.	To discover common characteristics across multiple modalities.	No	2 + is possible, 3 + is preferred	Features	Included inTensor ICA via ARD	The method provides a flexible framework for the fusion of multiple modalities with different units, signal- and contrast-to-noise ratios, voxel counts, spatial smoothnesses, and intensity distributions.
BigFLICA	Enhance Linked ICA by integrating mMIGP and DicL to deal with high-dimensional, large-sample datasets.	To discover common characteristics across multiple modalities from high-dimensional, large-sample multi-modal data features.	No	2 + is possible, 3 + is preferred	Features	mMIGP, DicL, Included inTensor ICA via ARD	The method can deal with multiple modalities from high-dimensional, large-sample multi-modal data features.
SuperBigFLICA	Minimize the reconstruction errors of the imaging data via BigFLICA and the prediction errors of nIDPs simultaneously.	To extract common characteristics across multiple modalities that exhibit predictive power for non-imaging derived phenotypes.	Yes	2 + is possible, 3 + is preferred	Features	mMIGP, DicL, Included inTensor ICA via ARD	The method takes advantage of BigFLICA and simultaneously predicts the nIDPs.
PICA	Maximize the independence of components for each modality separately based on the selected corresponding components where the mixing profile correlations across different modalities are above the threshold.	To identify both independent components and flexible connections among all modalities.	Yes	2 Is classical 3 + is possible	Features	PCA	The method provides a flexible framework to combine multiple data types with different ranges and properties, such as different neuroimaging, genetic, and phenotypic data.
pICA-R	Impose an additional constraint upon the infomax framework of pICA to minimize the distance between a certain component and the single reference.	To identify both independent components and flexible connections associated with the reference among all modalities.	Yes	2	Features	PCA	The method can discover correlated components that were significantly associated with the reference. However, pICA-R showed increased sensitivities with references of higher accuracies.
pICA-MR	Impose an additional constraint upon the infomax framework of pICA to minimize the distance between multiple components and multiple reference sets.	To identify both independent components and flexible connections associated with multiple references among all modalities.	Yes	2	Features	PCA	The method is flexible in dynamically identifying the constrained component for individual referential sets and allows some extent of heterogeneity in the reference.
Parallel group ICA + ICA	Integrate group ICA into pICA in a unified optimization framework, based on the selected corresponding components whose correlations between functional variability of group- and subject-specific spatial maps and mixing profile from the other modality are above the threshold.	To detect linked functional network variability and structural covariations and enable direct fusion of first-level fMRI features with another modality.	Yes	2	Features or raw data	PCA	The method can directly link first-level 4D fMRI data with another modality.
IVA	Assume statistical dependence across multiple datasets. Maximize the independence of each modality separately via ICA, define linked sources across datasets as source component vector (SCV), and maximize the independence among SCVs while considering the dependence across datasets.	To identify multiple linked sources among any number of modalities.	No	2 + is preferred	Features	No	The method provides a natural and extendable framework to directly link multiple modalities. However, it imposes a single structure to the data, where always a single component (none more, nor less) from each dataset must go together to form a subspace.
aNy-way ICA	Maximize the independence of each modality separately via ICA, and corresponding loadings are then organized into SCVs, followed by minimization of their mutual information with IVA.	To detect multiple linked sources over any number of modalities, and different numbers of sources for different modalities.	No	2 + is preferred	Features	No	The method provides a flexible framework to fuse datasets with any number of modalities, and different numbers of sources for different modalities.
C-ICT	Maximize the independence of each modality separately via ICA, select meaningful ICs and the corresponding mixing matrix, perform IVA on them to obtain SCVs and second-level mixing matrix, select SCVs with significant pair-wise correlations and trace back to the first stage to identify associated components across different modalities.	To discover one component from one modality linked with more than one component from another modality.	No	2 + is preferred	Features	No	The method is uniquely flexible in terms of the number of datasets, signal subspace order, and the opportunity to find “one-to-many associations.”
MISA	Decompose each modality into different sources, establish subspaces among groups of sources across different modalities to gauge their associations and pursue subspace independence, and finally recover linked features of flexible dimensionality from multiple modalities.	To capture shared and unique variability across and within modalities.	No	2 + is preferred	Features	No	The method combines modalities of different intrinsic dimensionality in a single model, and provides a direct, principled approach to multi-dataset combination.

Abbreviation: ICA, independent component analysis; jICA, joint ICA; pml-jICA, parallel multilink jICA; DS-ICA, disjoint subspace analysis using ICA; cmICA, connectivity matrix ICA; CCA, canonical correlation analysis; mCCA, multimodal/multi-set CCA; mCCAR + jICA, mCCA with reference + jICA; PM-SCCA, preference matrix guided sparse CCA; pICA, parallel independent component analysis; pICA-R, pICA with reference; pICA-MR, pICA with multiple references; BigFLICA, big-data linked ICA; SuperBigFLICA, Semi-supervised big-data linked ICA; IVA, independent vector analysis; MMIVA, multimodal IVA; C-ICT, consecutive independence and correlation transform; MISA, multidataset independent subspace analysis; nIDPs, non-imaging derived phenotypes; PCA, principal component analysis; ARD, automatic relevance determination; mMIGP, multimodal extension of MELODIC’s incremental group principal component analysis; DicL, dictionary learning.

## Review of multimodal fusion analysis in psychiatric disorders

We conduct a selective review of research to study associations among modalities in the context of psychiatric disorders with the previous data-driven multimodal fusion methods. Briefly, we searched PubMed for the terms multimodal, multimodal fusion, and multimodal modalities, and then narrowed these to studies that actually used one of the fusion-based approaches mentioned before. All of the multimodal fusion studies in psychiatric disorders reviewed in this study are summarized in Table 2. Generally speaking, most of the studies we reviewed demonstrate congruent effects across modalities, and multimodal fusion almost always provides more power to differentiate disease than unimodal approaches.

**Table 2:** Summary of multimodal MRI fusion applications in psychiatric disorders.

Method	Modality	Priors	Subject type	Studies
jICA	fMRI-sMRI	No	285 HC/89 SZ	(Antonucci *et al*., 2022)
	fMRI-sMRI-EEG	No	15 HC/15 SZ	(Calhoun *et al*., 2007)
	fMRI-sMRI	No	15 HC/15 SZ	(Calhoun, Adali, Giuliani, *et al*., 2006)
	dMRI-MEG	No	29 HC/29 SZ	(Stephen *et al*., 2013)
	fMRI-dMRI-sMRI	No	156 HC	(Yang *et al*., 2019)
	fMRI-sMRI	No	242 HC/220 SZ/147 SAD/180 SZ-BP	(Wang *et al*., 2015)
pml-jICA	fMRI-sMRI	No	130 HC/130 AD	(Khalilullah *et al*., 2023)
DS-ICA	fMRI-EEG	No	22 HC/16 SZ	(Adali *et al*., 2018)
	AOD1 task fMRI- AOD2 task fMRI	No	150 HC/121 SZ	(Akhonda *et al*., 2019)
	AOD task fMRI- SIRP task fMRI- SM task fMRI	No	138 HC/109 SZ	(Akhonda *et al*., 2021)
mCCA	fMRI-sMRI-EEG	No	53 HC/48 SZ	(Sui, Castro, *et al*., 2014)
	fMRI-dMRI-sMRI	No	50 HC/47 SZ	(Sui *et al*., 2015)
	fMRI-sMRI	No	23 HC/16 SZ	(Correa *et al*., 2008)
mCCA + jICA	fMRI-dMRI-sMRI	No	116 HC/97 SZ	(Sui, He, *et al*., 2012)
	fMRI-dMRI-sMRI	No	28 HC/35 SZ	(Sui *et al*., 2013)
	fMRI-sMRI	No	33 HC/40 MDD/13 BP	(He *et al*., 2017)
	fMRI-dMRI-sMRI	No	298 HC/307 SZ; 66 HC/40 SZ	(Liu *et al*., 2019)
	fMRI-sMRI	No	122 HC/89 EOS SZ; 34 HC/34 AOS SZ; 124 HC/126 chronic SZ	(Feng *et al*., 2022)
	dMRI-sMRI	No	30 HC/35 BP	(Tang *et al*., 2020)
	fMRI-sMRI	No	21 HC/19 SZ	(Lottman *et al*., 2018)
	dMRI-sMRI	No	34 HC/30 OCD	(Kim *et al*., 2015)
	fMRI-sMRI	No	55 HC/55 SZ	(Yao, Hu, *et al*., 2021)
	dMRI-sMRI	No	70 HC/99 MCI/62 SCD	(Liang *et al*., 2021)
	fMRI-sMRI	No	163 HC/151 SZ	(Abrol *et al*., 2017)
	fMRI-sMRI	No	31 HC/23 SZ/27 BP	(Lerman-Sinkoff *et al*., 2019)
	fMRI-sMRI	No	37 HC/37 SSD	(Hirjak *et al*., 2019)
	fMRI-sMRI	No	160 HC/150 SZ	(Duda *et al*., 2023)
	fMRI-dMRI-sMRI	No	298 HC/307 SZ	(Wang *et al*., 2019)
	fMRI-dMRI-sMRI	No	56 HC/90 SZ/37 BP/10 SAD	(DeRamus *et al*., 2022)
	dMRI-sMRI	No	41 HC/39 AD	(Ouyang *et al*., 2015)
	fMRI-dMRI	No	62 HC/54 SZ/48 BP	(Sui *et al*., 2011)
	fMRI-sMRI	No	160 HC/150 SZ	(Duda *et al*., 2022)
mCCAR + jICA	fMRI-dMRI-sMRI	Working memory	147 HC/147 SZ; 44 HC/39 SZ	(Qi, Calhoun, *et al*., 2018)
	fMRI-dMRI-sMRI	MicroRNA132	123 HC/81 MDD	(Qi, Yang*, et al*. 2018)
	fMRI-dMRI-sMRI	Cognition	147 HC/147 SZ; 44 HC/39 SZ; 42 HC–42 SZ	(Sui *et al*., 2018)
	fMRI-sMRI	Symptom severity	238 SZ/260 MDD/421 ASD 244 ADHD/313 drinkers/104 smokers	(Qi, Bustillo, *et al*., 2020)
	fMRI-sMRI	Symptom severity	229 ASD	(Qi, Morris, *et al*., 2020)
	fMRI-sMRI	Novelty seeking	1378 adolescents/147 SZ/81 MDD/320 ADHD/313 alcohol drinkers/104 smokers/1094 HC	(Qi *et al*., 2021)
	fMRI-sMRI	HDRS; cognition	54 MDD	(Qi, Calhoun, *et al*., 2022)
	fMRI-sMRI	SRS	72 HC/58 ASD; 23 HC/41 ASD	(Li *et al*., 2019)
	fMRI-sMRI	SZ PRS	22 459 HC	(Qi, Sui, *et al*., 2022)
PM-SCCA	PET-SNP	SNPs	237 HC/96 SMC/272 EMCI/ 225 LMCI/125 AD	(Sha *et al*., 2023)
Linked ICA	fMRI-sMRI-ASL	No	215 HC; 433 HC	(Liu, Tyler, *et al*., 2022)
	dMRI-sMRI	No	484 HC	(Groves *et al*., 2012)
	dMRI-sMRI	No	46 HC/46 ASD	(Itahashi *et al*., 2015)
	fMRI-dMRI-sMRI	No	71 HC/170 depression	(Maglanoc *et al*., 2020)
	dMRI-sMRI	No	93 HC/87 ADHD	(Wolfers *et al*., 2017)
	fMRI-dMRI-sMRI	No	119 HC/80 ADHD	(Wu *et al*., 2019)
BigFLICA	fMRI-dMRI-sMRI-swMRI-T2 FLAIR	No	1003 subjects (HCP); 14 503 subjects (UK Biobank)	(Gong *et al*., 2021)
SuperBigFLICA	fMRI-dMRI-sMRI-swMRI-T2 FLAIR	Target nIDP	39 770 subjects	(Gong *et al*., 2022)
pICA	fMRI-sMRI	Expert-knowledge-based threshold of correlation between components	47 HC/74 SZ	(Otte *et al*., 2023)
	fMRI-sMRI		19 HC/15 SZ-AVH/16 SZ-nAVH	(Kubera *et al*., 2019)
	fMRI-sMRI-SNP		87 HC/20 first-episode SZ/52 chronic SZ	(Luo *et al*., 2019)
	dMRI-sMRI		82 HC/73 SZ	(Jensen *et al*., 2022)
	fMRI-SNP		87 HC/61 SZ	(Rashid *et al*., 2019)
	sMRI-SNP		209 HC/367MCI/181 AD	(Meda *et al*., 2012)
	fMRI-sMRI		162 HC/149 SZ	(Qi *et al*., 2019)
IVA	AOD task fMRI- SIRP task fMRI- SM task fMRI	No	150 HC/120 SZ	(Levin-Schwartz *et al*., 2017)
	AOD task fMRI- SIRP task fMRI- SM task fMRI	No	38 HC/109 SZ	(Akhonda *et al*., 2022)
	VIS task fMRI- AOD task fMRI- SM task fMRI	No	150 HC/121 SZ	(Luo *et al*., 2020)
MMIVA	fMRI-dMRI-sMRI	No	3497 subjects	(Damaraju *et al*., 2021)
C-ICT	fMRI-dMRI-sMRI	No	86 HC/76 SZ	(Jia *et al*., 2021)

Abbreviations: ICA, independent component analysis; jICA, joint ICA; pml-jICA, parallel multilink jICA; DS-ICA, disjoint subspace analysis using ICA; CCA, canonical correlation analysis; mCCA, multimodal/multi-set CCA; mCCAR + jICA, mCCA with reference + jICA; PM-SCCA, preference matrix guided sparse CCA; pICA, parallel ICA; BigFLICA, big-data linked ICA; SuperBigFLICA, Semi-supervised big-data linked ICA; IVA, independent vector analysis; MMIVA, multimodal IVA; C-ICT, consecutive independence and correlation transform; HC, healthy control; SZ, schizophrenia; SAD, schizoaffective disorder; BP, bipolar disorder; MDD, major depressive disorder; SSD, schizophrenia spectrum disorder; AD, Alzheimer's disease; MCI, mild cognitive impairment; ; SMC: subjective memory complaint; EMCI: early mild cognitive impairment; LMCI: late mild cognitive impairment; ADHD, attention-deficit/hyperactivity disorder; ASD, autism spectrum disorder; OCD, obsessive-compulsive disorder; SCD, subjective cognitive decline; EOS, adolescent early-onset; AOS, adult-onset; AVH, auditory verbal hallucination; HDRS, Hamilton Depression Rating Scale; SRS, Social Responsiveness Scale; PRS, polygenic risk scores.

### Schizophrenia

#### Blind multimodal fusion

**Figure 2:**
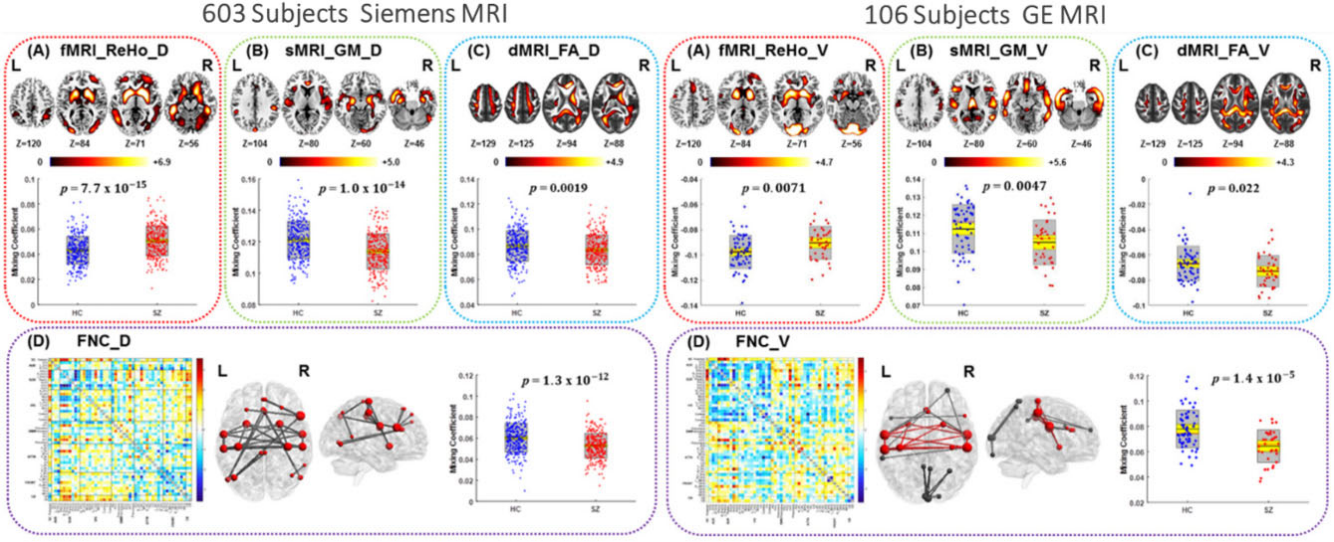
The application of blind four-way multiset CCA plus jICA to identify multimodal alteration in SZ. Covarying functional and structural abnormalities were identified in (**A**) regional homogeneity (ReHo), (**B**) GM, (**C**) FA, and (**D**) functional network connectivity (FNC) in two independent cohorts using different scanners, where the spatial maps of ReHo, GM, and FA were visualized at |*Z*| >2.5 with the positive *Z* scores shown in red, and the FNC matrix was transformed into *Z* scores and thresholded at |*Z*| >3. Reproduced with permission from Liu *et al*. (2019).

Numerous studies have demonstrated that blind multimodal fusion can capture the co-occurring abnormalities in brain function and structure in patients with schizophrenia (SZ). Antonucci *et al*. identified aberrant structural-functional covariation networks using jICA (Antonucci *et al*., 2022), showing significantly reduced covariation between temporoparietal degree centrality and GM volume (GMV) in frontal, temporal, parietal cortex, and thalamus in SZ patients, which was also associated with both social and occupational functioning. However, no group difference was found in degree centrality using univariate analysis, demonstrating that leveraging the cross-information among multiple imaging modalities may provide meaningful results. One study combined fMRI, dMRI, and sMRI by mCCA on a dataset from 47 SZ and 50 healthy controls (HC) to identify covarying patterns of fALFF, FA, and GMV. One multimodal component was identified as both group discriminating and significantly correlated with the MATRICS Consensus Cognitive Battery composite (Sui *et al*., 2015). A main finding was that linked functional and structural deficits in the distributed cortico-striatal-thalamic circuit may account for several aspects of cognitive impairment in SZ. Particularly, results found that distinct dimensional aspects of cognitive composite might exhibit dissociable multimodal imaging signatures, as the increased fALFF values in the inferior parietal lobule significantly correlated with declined social cognition. Similarly, Sui *et al*. integrated ALFF, EEG spectra, and GM using mCCA to distinguish SZ from HC with >90% classification accuracy (Sui, Castro, *et al*., 2014). In addition, using four types of MRI feature in a joint analysis to investigate multiple impairments of SZ on a large population (Fig. 2), researchers not only identified covarying functional and structural regions in the striatum, hippocampus, and frontal-parietal network, but also found high spatial consistency of these altered regions across different scanners using mCCA + jICA (Liu *et al*., 2019). This suggests that the fusion results of mCCA + jICA are highly robust and replicable, while offering unique perspectives regarding the missing links between modalities.

Using tIVA, a more flexible fusion approach, Adali *et al*. identified significant group difference in the covariation of fMRI and EEG between SZ and HC, but not in all fMRI, sMRI, and EEG modalities (Adali *et al*., 2015). Significant group differences were found in the temporal-motor activation in fMRI and the N2 peak in EEG. Moreover, not limiting to the one-to-one fusion patterns, the study combined FA, GM, and fALFF for SZ and HC with C-ICT, identifying six interpretable triplets of components, each of which consists of three associated components from the three modalities (Jia *et al*., 2021). For instance, the corticospinal tract and superior longitudinal fasciculus from dMRI were not only associated with the uncus and inferior temporal gyrus from sMRI and superior frontal gyrus and middle frontal gyrus from fMRI, but were also associated with the precuneus and paracentral lobule from sMRI and superior temporal gyrus from fMRI. This indicates that C-ICT can reveal multiple associations across three modalities and provide potential biomarkers for SZ, and is a flexible and informative method for the fusion of medical imaging data from different modalities.

#### Semi-blind multimodal fusion

**Figure 3:**
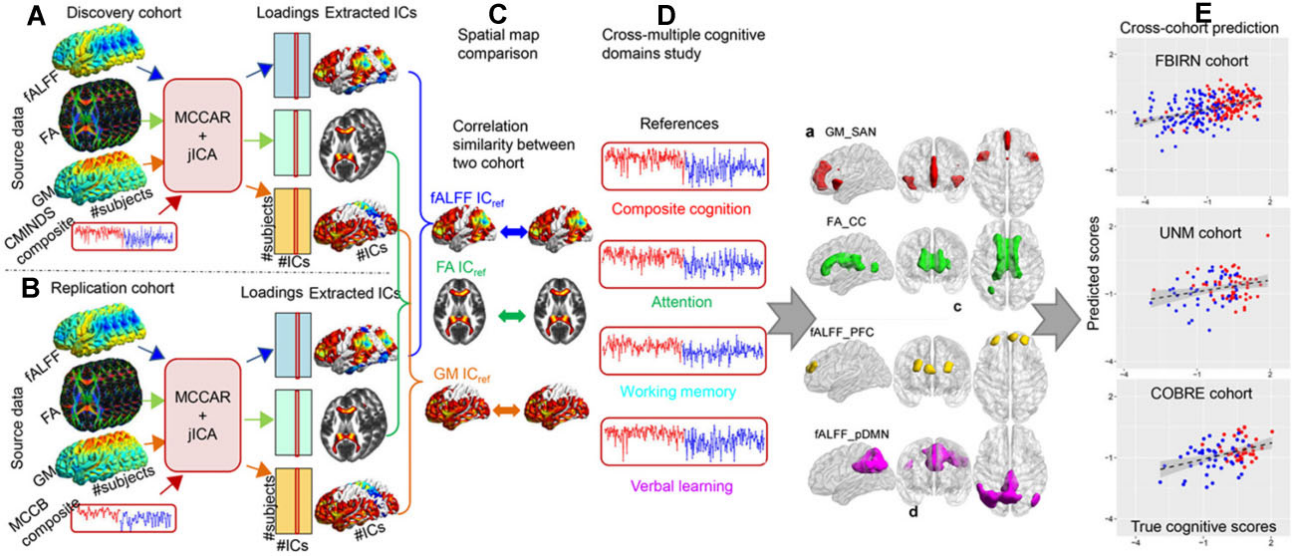
Cognition-directed multimodal fusion and prediction analysis using multi-site CCA with reference plus jICA (mCCAR + jICA). Cognition-associated multimodal covarying imaging patterns were identified in three modalities and are highly consistent across cohorts. More importantly, the identified imaging signatures are predictive of multiple-domain cognitive performance. Reproduced with permission from Sui *et al*. (2018).

By introducing prior information, semi-blind multimodal fusion approaches enhance the sensitivity and specificity of identifying meaningful brain imaging covariance patterns that are associated with specific symptoms, cognitive deficits, and gene expression changes observed in psychiatric disorders. As shown in Fig. 3, cognitive global scores were used to guide three-way multimodal MRI fusion in two independent cohorts including both HC and SZ via the supervised learning strategy with mCCAR + jICA. The findings suggested that the salience network in GM, corpus callosum in FA, and central executive and default-mode networks in fALFF can serve as modality-specific biomarkers of generalized cognition (Sui *et al*., 2018). The identified MRI signatures are highly consistent cross-cohort and, more importantly, they are predictive of multiple-domain cognitive performance, suggesting that the reference-guided multimodal fusion results may serve as effective predictors for the relevant cognitive measures (Sui *et al*., 2018).

**Figure 4:**
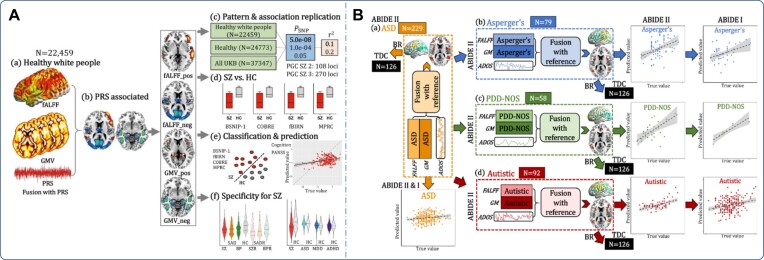
(**A**) Multimodal covarying analysis guided by the PRS for SZ using mCCAR + jICA. The study identified that the SZ-PRS was associated with decreased GMV and fALFF in the frontotemporal cortex, which can distinguish SZ from HCs with more than 83% accuracy, and can significantly predict their cognition and symptoms across four independent cohorts. More interestingly, the study found that the identified frontotemporal alterations were specific to SZ. (**B**) Multimodal covarying analysis guided by autistic symptom score for ASD and its three subtypes using mCCAR + jICA. The study showed that the dorsolateral prefrontal cortex and superior/middle temporal cortex in fALFF and GM are the shared covarying regions among the three subtypes, while the key differences among the three subtypes are negative functional features within subcortical brain areas. Moreover, each subtype-specific brain pattern is correlated with different symptom subdomains, with social interaction as the common subdomain. Reproduced with permission from Qi, Morris, *et al*. (2020) and Qi, Sui, *et al*. (2022).

Similarly, Qi *et al*. used mCCAR + jICA to investigate the fMRI-sMRI covarying patterns associated with the polygenic risk scores (PRS) for SZ on the UK Biobank dataset (Qi, Sui, *et al*., 2022). Results showed a robust PRS-associated neuroimaging pattern with decreased GMV and fALFF in the frontotemporal cortex, which can distinguish SZ from HC with >83% accuracy and can significantly predict their cognition and symptoms across four independent cohorts (Fig. 4A). More interestingly, the identified frontotemporal alterations were found to be impaired in patients with schizoaffective disorder (SAD), but not in autism spectrum disorder (ASD), depression, and attention-deficit/hyperactivity disorder (ADHD), suggesting the potential multimodal brain biomarker specific to SZ.

### Mood disorders

#### Blind multimodal fusion

One study used multimodal fusion to investigate mood disorders, jointly analyzing fMRI and sMRI via mCCA + jICA in major depressive disorder (MDD), bipolar disorder (BP), and HC (He *et al*., 2017). The group discriminative covarying components were identified with reduced GM in the parietal and occipital cortices, and attenuated functional connectivity within sensory and motor networks for BP patients compared with HC, while showing the altered GM in the amygdala and cerebellum for MDD patients. In contrast to unimodal data, the identified multimodal patterns can distinguish MDD, and BP from HC with higher classification accuracy. Similarly, Tang *et al*. used mCCA + jICA to investigate the sMRI-dMRI covarying patterns in BP patients (Tang *et al*., 2020). One multimodal covarying pattern was identified with decreased GM in the inferior frontal gyrus, right anterior cingulate gyrus and left superior frontal gyrus, which was associated with reduced WM integrity in the corticospinal tract and superior longitudinal fasciculus.

#### Semi-blind multimodal fusion

One study investigated how miR-132 dysregulation may affect covariation of multimodal brain imaging data in 81 unmedicated MDD patients and 123 demographically matched HCs using mCCAR + jICA, as well as in a medication-naive subset of MDD patients (Qi, Yang, *et al*., 2018). The findings suggested that higher miR-132 levels in MDD were associated with both lower fALFF and lower GMV in the fronto-limbic network. Moreover, the identified brain regions linked with increased miR-132 levels were also associated with poorer cognitive performance in attention and executive function. From the aspect of electroconvulsive therapy treatment (ECT) response, Qi *et al*. performed a 17-item Hamilton Depression Rating Scale guided brain structure-function fusion analysis via mCCAR + jICA in 118 patients with depressive episodes and 60 HCs (Qi, Abbott, *et al*., 2020). Results demonstrated that higher ECT responsiveness was associated with reduced fALFF in the prefrontal cortex, insula, and hippocampus, linked with increased GMV in the anterior cingulate, medial temporal cortex, insula, thalamus, caudate, and hippocampus. Relative to non-responders, responder-specific ECT-related brain networks occur in the fronto-limbic network and are associated with successful therapeutic outcomes. Although ECT is recommended as an efficacious therapy for treatment-resistant depression, patients often experience cognitive impairment after ECT treatment (Semkovska *et al*., 2010). One recent study combined fMRI and sMRI to identify ECT antidepressant-response and cognitive-impairment multimodal brain networks by mCCAR + jICA (Qi, Calhoun, *et al*., 2022). The findings exhibited decreased fALFF in the superior orbitofrontal cortex and caudate accompanied by increased GMV in the medial temporal cortex in both antidepressant-response and cognitive-impairment networks. For the modality-specific components, increased GMV in the hippocampus and thalamus were specific to antidepressant response, while decreased fALFF in the amygdala and hippocampus was specific to antidepressant response, which was validated in two independent datasets. More interestingly, the E-field within these two networks showed an inverse relationship with depressive symptom reduction and cognitive impairment, and the optimal E-fled range as [92.7–113.9] V/m was estimated to maximize antidepressant outcomes without compromising cognitive safety, which may improve the ECT benefit to risk ratio. All these studies indicate the superiority of the supervised multimodal fusion approaches in identifying potential biomarkers linked to specific symptoms, gene expression, and personalized treatment optimization.

### Transdiagnostic research

#### Blind multimodal fusion

Lifetime comorbidity among psychiatric disorders is pervasive, such as SZ and BP, and multimodal fusion allows us to leverage multimodal data to explore common and specific mechanisms of multiple psychiatric disorders (Buckley *et al*., 2009), which may contribute to the early diagnosis and treatment for specific disorders. Using jICA, Wang *et al*. investigated aberrant interactions between structure and function across SZ, SAD, and BP (Wang *et al*., 2015), showing that the common alterations across psychotic diagnoses were the covariations between ALFF in prefrontal-striatal-thalamic-cerebellar networks and GM in the DMN, which were also correlated with cognitive function, social function, and Schizo-Bipolar Scale scores, whereas the fused alteration in the temporal lobe was unique to SZ and SAD.

#### Semi-blind multimodal fusion

For multiple psychiatric disorders, the study combined fMRI and sMRI to explore symptom-driven transdiagnostic shared networks between SZ and substance use with drinking, smoking, depression, ASD, and ADHD via multi-group data mining (Qi, Bustillo, *et al*., 2020). Results demonstrated that substance use was associated with cognitive deficits in SZ through the anterior cingulate cortex and thalamus in GMV; that depression was linked to the negative dimensions of the positive and negative syndrome scale and reasoning in SZ through caudate-thalamus-middle/inferior temporal gyrus in GMV; and that developmental disorder pattern was correlated with poor attention, speed of processing, and reasoning in SZ through inferior temporal gyrus in GMV, indicating that distinct comorbid psychiatric conditions are accompanied by distinct impaired brain networks associated with different symptoms and cognitive impairments. Moreover, Qi *et al*. combined three fMRI tasks and sMRI to explore the multimodal covarying patterns associated with novelty seeking on the IMAGEN dataset. Results identified a covarying pattern including the prefrontal cortex, striatum, amygdala, and hippocampus, which can longitudinally predict five different risk scales, including alcohol drinking, smoking, hyperactivity, depression, and SZ disorders, and can also classify among ADHD, depression, and SZ with an accuracy of 87.2%, revealing a potential transdiagnostic neuroimaging biomarker to predict disease risks or severity.

### Other psychiatric disorders

#### Blind multimodal fusion

One study combined fMRI, dMRI, and sMRI to explore ADHD using linked ICA (Wu *et al*., 2019), suggesting that children with ADHD showed altered white matter microstructure in widespread white matter fiber tracts, increased GMV in bilateral frontal regions, and decreased GMV in posterior regions, as well as altered FC in default mode and frontoparietal networks. Wolfers *et al*. found the most predictive multimodal region for adult ADHD was primarily located in the anterior temporal cortex by combining dMRI and sMRI using linked ICA (Wolfers *et al*., 2017). In addition, one recent study used the pml-jICA to fuse sMRI and fMRI in Alzheimer's disease patients (Khalilullah *et al*., 2023), identifying two joint components with partially overlapping regions that showed opposite effects for Alzheimer's disease versus controls, but were able to be separated due to being linked to distinct functional and structural patterns.

#### Semi-blind multimodal fusion

There is a large heterogeneity in ASD, and one recent study combined GM and fALFF to dissect the heterogeneity in ASD by mCCAR + jICA with the Autism Diagnostic Observation Schedule as a reference to guide multimodal fusion on Asperger's, pervasive developmental disorder-not otherwise specified (PDD-NOS), and autistic subtype from the ABIDE I/II datasets (Qi, Morris, *et al*., 2020). Results showed that the dorsolateral prefrontal cortex and superior/middle temporal cortex were the primary common functional-structural covarying cortical brain areas shared among the three subtypes, while the key differences among the three subtypes were negative functional features within subcortical brain areas (Fig. 4B). Moreover, each subtype-specific brain pattern was correlated with different Autism Diagnostic Observation Schedule subdomains, with social interaction as the common subdomain.

Despite this study reviewing the application of multimodal fusion in psychiatric disorders, the data-driven fusion approaches have also been successfully applied in other diseases, such as human immunodeficiency virus disease (Sui *et al*., 2021), epilepsy (Zhi *et al*., 2020), and substance use disorders (Vergara *et al*., 2014; Hirjak *et al*., 2022), to reveal potential multimodal imaging biomarkers.

## Emerging trends

Substantial progress has been made in multimodal fusion approaches, from jICA constrained with the same mixing matrix and order of components as well as equal contributions for different modalities to mCCA, mCCA(R) + jICA, linked ICA, pICA, and IVA and its variants with more flexible framework to combine multiple data types, from identifying one-to-one to one-to-many associations, from identifying covarying components to discovering covarying and modality-specific components simultaneously among multiple modalities. However, there still remains much work to be done. With the collection of large-scale datasets, such as the UK Biobank with half a million UK participants, and various data types, such as gene, environment, transcriptome array, and behavior, the question of how to effectively fuse various types of data to identify potential stable and generalizable imaging biomarkers from high-dimensional data for psychiatric disorders remains. Additionally, deep learning, which can handle nonlinear features and learn high-dimensional representations, has been overwhelmingly successful in computer vision, natural language processing, and video/speech recognition (LeCun *et al*., 2015). Combining advanced deep learning approaches with the unique characteristics of brain imaging data is also a promising avenue for multimodal brain imaging analysis. Ultimately, quantitative multimodal fusion research needs to pay more attention to clinical translation, including the early diagnosis of high-risk populations and personalized treatment.

### N-way multimodal fusion

The release of large-scale datasets, such as the UK Biobank, ABCD, and HCP datasets, presents an unprecedented opportunity to mine complementary information from diverse modalities. Several studies have developed data-driven multimodal fusion approaches to combine multiple modalities on larger datasets. For example, BigFLICA was developed to integrate 47 imaging modalities to predict thousands of phenotypic and behavioral variables on the UK Biobank and HCP datasets, ~20 times faster than linked ICA while achieving improved predictive power compared with widely used analysis strategies, single-modality decompositions (Gong *et al*., 2021). Furthermore, by incorporating prior information, SuperBigFLICA was proposed to leverage multiple imaging modalities to predict phenotypes, and has been performed on the UK Biobank dataset with ~40 000 participants and 47 imaging modalities, along with >7000 non-imaging derived phenotypes. Results showed that the SuperBigFLICA approach improved the prediction accuracy of phenotypes by up to 46% compared to conventional expert-knowledge and unsupervised-learning approaches. Additionally, this approach can also learn the generic imaging features that can predict new phenotypes (Gong *et al*., 2022). In addition, Damaraju *et al*. performed multimodal IVA on a large multimodal dataset of >3000 participants in the UK Biobank study to identify GM-FA-ALFF linked independent sources, capturing age-associated covarying biomarkers with GM in thalamus, caudate, and insular regions, as well as FA in periventricular and ALFF in visual and parietal regions (Damaraju *et al*., 2021). More advanced models, such as those that can handle N-way multimodal fusion, are being introduced and may become one of the leading directions in future neuroimaging research given the predominance of multimodal data acquisition. Additionally, while the integration of large datasets and multimodal analyses offers promising opportunities to advance psychiatric research, challenges related to data quality, harmonization, statistical methods, and interpretation must be carefully addressed.

### Deep learning

**Figure 5:**
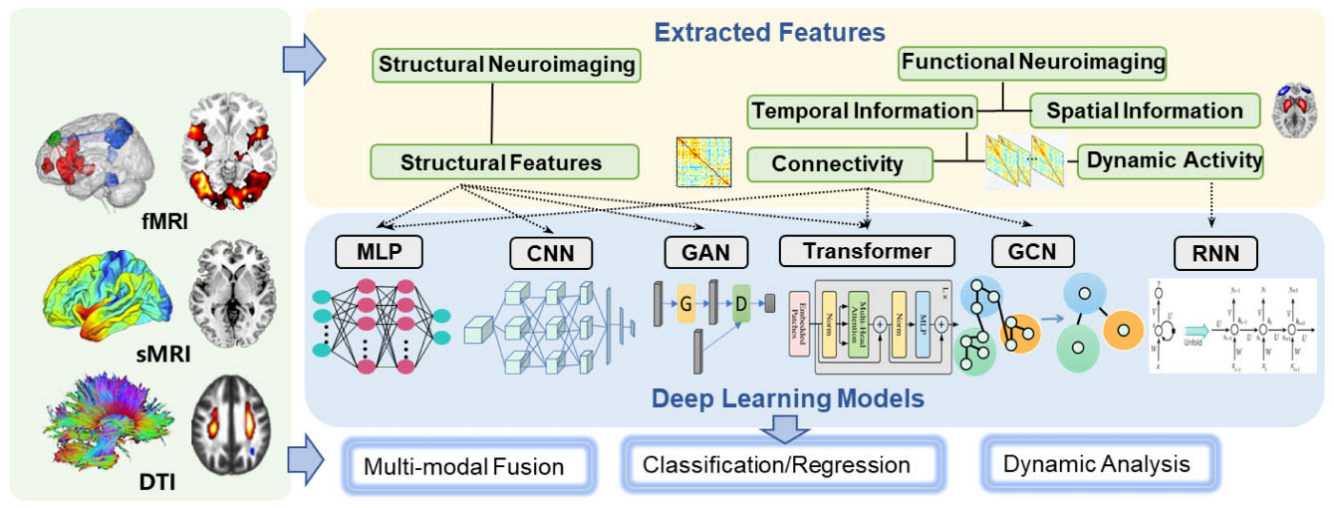
Deep learning frameworks that are popularly adopted by diverse MRI features.

Deep learning has emerged as a powerful and transformative approach in the field of medical brain imaging research, including convolutional neural networks for capturing spatial patterns, recurrent neural networks for handling time-series data, and graph convolutional networks for extracting topological properties, achieving unprecedented accuracy in disease diagnosis and image segmentation (Bzdok *et al*., 2018; Yan *et al*., 2022; Rahaman *et al*., 2023). Deep learning can automatically extract meaningful features from diverse neuroimaging modalities, such as sMRI, fMRI, and PET scans, to learn intricate variations and subtle abnormalities, thereby improving classification or predictive accuracy for various neurological and psychiatric disorders, as shown in Fig. 5.

For instance, an enhanced multi-modal graph convolutional network was constructed by fusing the brain structural and functional graphs to distinguish HC from neuropsychiatric disorders, including SZ, BP, and ADHD, achieving a classification accuracy of 93.71% (Liu, Wang, *et al*., 2022). A mutual multi-scale triplet graph convolutional network that combined functional and structural connectivity enhanced the accuracy for multiple brain disorder classification (Yao, Sui, *et al*., 2021). In medical imaging, interpretability in deep learning is not merely a desirable feature but an indispensable necessity (Bi *et al*., 2023). One recent study developed an interpretable multimodal fusion framework by combining intermediate feature maps with gradient-based weights, which can perform automated diagnosis and result interpretation simultaneously (Hu *et al*., 2021). These findings suggest that deep learning could provide more accurate and early detection of brain abnormalities (Zhang *et al*., 2011; Li *et al*., 2020; Zhao *et al*., 2022), which may not have been revealed through separate unimodal analyses as typically performed in most neuroimaging experiments.

### Clinical translation

The overarching goal of multimodal brain image fusion analysis for psychiatric disorders is to assist in clinical diagnosis and treatment. There is an increasing number of studies demonstrating the potential fusion of structural and functional data to improve brain disease classification and predictions (Gao *et al*., 2018; Lalousis *et al*., 2021; Wen *et al*., 2021; Xu *et al*., 2021; Zhi *et al*., 2021). For example, Sui *et al*. combined resting-state fMRI, EEG and sMRI data to classify 48 SZ from 53 HC and achieved the best performance with 91% accuracy compared to each single modality, confirming the effectiveness and advantages of multimodal fusion (Sui, Castro, *et al*., 2014). However, the classification or prediction accuracy tends to be relatively low with the increased sample size, especially for ADHD, autism, and depression, hindering the translation of research evidence into clinical practice (Woo *et al*., 2017). One future direction lies in building individualized prediction models based on imaging features derived from multimodal fusion, and combining behavioral, environmental, or genetic variants to improve the accuracy of psychiatric diagnosis, risk warning, or treatment optimization (Jiang *et al*., 2018; Jiang *et al*., 2020; Sui *et al*., 2020; Qi *et al*., 2021).

## Conclusions

In summary, this selective review underscores the pivotal role of data-driven multimodal fusion approaches in advancing our understanding of psychiatric disorders and revolutionizing psychiatric research. By leveraging various data types, such as different imaging modalities and phenotypes, these approaches have shown great promise in the identification of individualized signatures associated with clinical symptoms, personalized diagnosis, or intervention parameters. With the ever-expanding collection of rich information encompassing gene expression, environmental exposures, protein expression, imaging features, and behavioral outcomes, the most promising avenues for the future may lie in developing better data mining models that can complement and harness the intricate relationships between diverse neuroimaging and other forms of data, and ultimately translating scientific discoveries into meaningful clinical translation.

## Code availability

The codes for the jICA, pICA, mCCA, mCCA + jICA, mCCAR + mICA, parallel group ICA + ICA, and IVA methods are all available in the fusion ICA toolbox (FIT, http://trendscenter.org/software/fit). The codes for other fusion methods are available on various websites including the Linked ICA (https://fsl.fmrib.ox.ac.uk/fsl/fslwiki/FLICA), MISA (https://github.com/rsilva8/MISA), BigFLICA (https://github.com/weikanggong/BigFLICA), and SuperBigFLICA (https://github.com/weikanggong/SuperBigFLICA).
